# 2,3-Dibromo-1-(4-methyl­phen­yl)-3-(5-nitro­furan-2-yl)propan-1-one

**DOI:** 10.1107/S1600536810050488

**Published:** 2010-12-11

**Authors:** Hoong-Kun Fun, Tara Shahani, Nithinchandra, Balakrishna Kalluraya

**Affiliations:** a X-ray Crystallography Unit, School of Physics, Universiti Sains Malaysia, 11800 USM, Penang, Malaysia; b Department of Studies in Chemistry, Mangalore University, Mangalagangotri, Mangalore 574 199, India

## Abstract

In the title compound, C_14_H_11_Br_2_NO_4_, the whole mol­ecule is disordered over two positions with a refined occupancy ratio of 0.539 (9):0.461 (9). The 2-nitro­furan and toluene groups are approximately planar, with maximum deviations of 0.176 (11) and 0.121 (14) Å, respectively, in the major component and 0.208 (11) and 0.30 (17) Å in the minor component. The dihedral angles between the 2-nitro­furan and toluene groups are 8.7 (5) and 8.0 (9)° for the major and minor components, respectively. In the crystal, weak inter­molecular C—H⋯O inter­actions connect mol­ecules into a three-dimensional network, generating *R*_2_^1^(6) ring motifs.

## Related literature

For the biological activity of nitrofurans, see: Holla *et al.* (1986 ▶, 1987 ▶, 1992 ▶); Hegde *et al.* (2006 ▶); Rai *et al.* (2008 ▶). For a related structure, see: Fun *et al.* (2010 ▶). For the stability of the temperature controller used in the data collection, see: Cosier & Glazer (1986 ▶). For standard bond-length data, see: Allen *et al.* (1987 ▶). For hydrogen-bond motifs, see: Bernstein *et al.* (1995 ▶).
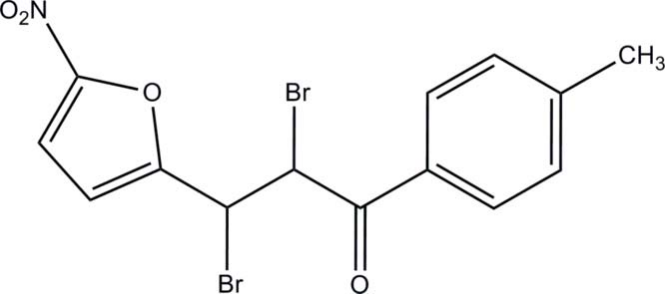


## Experimental

### 

#### Crystal data

C_14_H_11_Br_2_NO_4_
*M* *_r_* = 417.06
Triclinic, 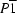

*a* = 8.7766 (3) Å
*b* = 9.0386 (3) Å
*c* = 10.4841 (3) Å
α = 87.601 (2)°
β = 75.505 (2)°
γ = 69.554 (2)°
*V* = 753.53 (4) Å^3^
*Z* = 2
Mo *K*α radiation
μ = 5.39 mm^−1^
*T* = 100 K
0.47 × 0.21 × 0.13 mm

#### Data collection

Bruker APEXII DUO CCD area-detector diffractometer
Absorption correction: multi-scan (*SADABS*; Bruker, 2009 ▶) *T*_min_ = 0.184, *T*_max_ = 0.550
10357 measured reflections
3465 independent reflections
2729 reflections with *I* > 2σ(*I*)
*R*_int_ = 0.026

#### Refinement

*R*[*F* ^2^ > 2σ(*F* ^2^)] = 0.041
*wR*(*F* ^2^) = 0.103
*S* = 1.19
3465 reflections
274 parameters
658 restraints
H-atom parameters constrained
Δρ_max_ = 0.88 e Å^−3^
Δρ_min_ = −0.40 e Å^−3^

### 

Data collection: *APEX2* (Bruker, 2009 ▶); cell refinement: *SAINT* (Bruker, 2009 ▶); data reduction: *SAINT*; program(s) used to solve structure: *SHELXTL* (Sheldrick, 2008 ▶); program(s) used to refine structure: *SHELXTL*; molecular graphics: *SHELXTL*; software used to prepare material for publication: *SHELXTL* and *PLATON* (Spek, 2009 ▶).

## Supplementary Material

Crystal structure: contains datablocks global, I. DOI: 10.1107/S1600536810050488/lh5178sup1.cif

Structure factors: contains datablocks I. DOI: 10.1107/S1600536810050488/lh5178Isup2.hkl

Additional supplementary materials:  crystallographic information; 3D view; checkCIF report

## Figures and Tables

**Table 1:** Hydrogen-bond geometry (Å, °)

*D*—H⋯*A*	*D*—H	H⋯*A*	*D*⋯*A*	*D*—H⋯*A*
C2*A*—H2*AA*⋯O3*A*^i^	0.93	2.53	3.210 (15)	131
C3*A*—H3*AA*⋯O2*A*^ii^	0.93	2.51	3.216 (12)	133
C6*A*—H6*AA*⋯O2*A*^ii^	0.98	2.33	3.217 (10)	151
C13*A*—H13*A*⋯O3*A*^iii^	0.93	2.55	3.434 (13)	158

Symmetry codes: (i) 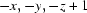; (ii) 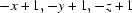; (iii) 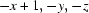.

**Fig. 1.**
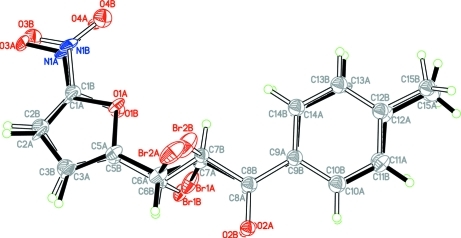
The molecular structure of the title compound, showing 50% probability displacement ellipsoids and the atom-numbering scheme. The minor component of disorder is shown with open bonds.

**Fig. 2.**
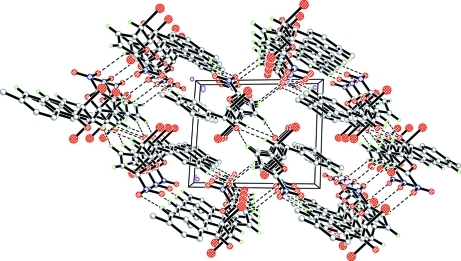
The crystal packing of the title compound, viewed along a axis. Only the major disordered component is shown. Hydrogen atoms not involved in hydrogen boding are omitted for clarity.

## supplementary crystallographic information

### Comment

Nitrofurans are a class of synthetic compounds characterized by the presence of 5-nitro-2-furyl group. The presence of nitro group in position-5 of the molecule conferred antibacterial activity (Holla *et al.*1986). A number of nitrofurans have attained commercial utility as antibacterial agents in humans and in veterinary medicine because of their broad spectrum of activities (Holla & Kalluraya *et al.*, 1992; Holla *et al.*, 1987). The incorporation of 5-nitrofuran or 5-nitrothiophene moiety into various heterocyclic systems has found to increase their biological activities. We have reported few heterocyclic systems carrying a 5-nitrofuran moiety as potent antimicrobial agents (Hegde *et al.*, 2006). During the synthetic procedures, the dibromopropanones were obtained by the bromination of 1-aryl-3-(5-nitro-2-furyl)-2-propen-1-ones. Acid-catalysed condensation of acetophenones with nitrofural diacetate in acetic acid yielded the required 1-aryl-3-(5-nitro-2-furyl)-2-propen-1-ones (chalcones) (Rai *et al.*, 2008).

In the title compound (Fig. 1), the whole molecule is disordered over two positions with a refined occupancy ratio of 0539 (9):0.461 (9) The molecule consists of a 2-nitrofuran (C1–C3/C5/N1/O1/O3/O4) group, a toluene group which (C9–C15) and one 2, 3-dibromopropanal (C6–C9/Br1/Br2/O2) moiety. Both ring groups are essentially planar (maximum deviation of 0.176 (11) and 0.121 (14) Å in the major component and 0.208 (11) and 0.30 (17) Å in the minor component for the 2-nitrofuran and toluene groups respectively). The bond lengths (Allen *et al.*, 1987) and angles are within normal ranges and are comparable to a closely related structure (Fun *et al.*, 2010).

In the crystal packing (Fig. 2), intermolecular C3A—H3A···O2A^ii^ and C6A—H6A···O2A^ii^ hydrogen bonds connect neighbouring molecules generating *R*_2_^1^(6) ring motifs (Bernstein *et al.*, 1995) (Table 1). These dimers are linked into a three-dimensional network by intermolecular C2A—H2AA···O3A^i^ and C13A—H13A···O3A^iii^ hydrogen bonds (Table 1).

### Experimental

1-(4-Methylphenyl)-3-(5-nitro-2-furyl)-2-propen-1-one (0.01 mol) was dissolved in glacial acetic acid (25 ml) by gentle warming. A solution of bromine in glacial acetic acid (30%*w*/*v*) was added to it with constant stirring till the yellow color of the bromine persisted. The reaction mixture was kept aside at room temperature for overnight. Crystals of dibromopropanones which separated out were collected by filtration and washed with ethanol and dried and then recrystallized from glacial acetic acid. Crystals suitable for X-ray analysis were obtained from 1:2 mixtures of DMF and ethanol by slow evaporation.

### Refinement

All the H atoms were positioned geometrically [C–H = 0.93 to 0.98 Å] and were refined using a riding model, with *U*_iso_(H) = 1.2 *U*_eq_ (C). The whole molecule is disordered over two positions with a refined ratio of 0539 (9):0.461 (9) Initially rigid, similarity and simulation restraints were applied. After steady state has been reached, rigid restraints were removed for the final refinement.

### Crystal data

**Table:**

C_14_H_11_Br_2_NO_4_	*Z* = 2
*M**_r_* = 417.06	*F*(000) = 408
Triclinic, *P*1	*D*_x_ = 1.838 Mg m^−^^3^
Hall symbol: -P 1	Mo *K*α radiation, λ = 0.71073 Å
*a* = 8.7766 (3) Å	Cell parameters from 4461 reflections
*b* = 9.0386 (3) Å	θ = 2.8–29.8°
*c* = 10.4841 (3) Å	µ = 5.39 mm^−^^1^
α = 87.601 (2)°	*T* = 100 K
β = 75.505 (2)°	Block, colourless
γ = 69.554 (2)°	0.47 × 0.21 × 0.13 mm
*V* = 753.53 (4) Å^3^	

### Data collection

**Table:**

Bruker APEXII DUO CCD area-detector diffractometer	3465 independent reflections
Radiation source: fine-focus sealed tube	2729 reflections with *I* > 2σ(*I*)
graphite	*R*_int_ = 0.026
φ and ω scans	θ_max_ = 27.5°, θ_min_ = 2.0°
Absorption correction: multi-scan (*SADABS*; Bruker, 2009)	*h* = −11→11
*T*_min_ = 0.184, *T*_max_ = 0.550	*k* = −11→11
10357 measured reflections	*l* = −13→13

### Refinement

**Table:**

Refinement on *F*^2^	Primary atom site location: structure-invariant direct methods
Least-squares matrix: full	Secondary atom site location: difference Fourier map
*R*[*F*^2^ > 2σ(*F*^2^)] = 0.041	Hydrogen site location: inferred from neighbouring sites
*wR*(*F*^2^) = 0.103	H-atom parameters constrained
*S* = 1.19	*w* = 1/[σ^2^(*F*_o_^2^) + (0.0366*P*)^2^ + 1.118*P*] where *P* = (*F*_o_^2^ + 2*F*_c_^2^)/3
3465 reflections	(Δ/σ)_max_ < 0.001
274 parameters	Δρ_max_ = 0.88 e Å^−^^3^
658 restraints	Δρ_min_ = −0.40 e Å^−^^3^

### Special details

**Table:**

Experimental. The crystal was placed in the cold stream of an Oxford Cyrosystems Cobra open-flow nitrogen cryostat (Cosier & Glazer, 1986) operating at 100.0 (1) K.
Geometry. All e.s.d.'s (except the e.s.d. in the dihedral angle between two l.s. planes) are estimated using the full covariance matrix. The cell e.s.d.'s are taken into account individually in the estimation of e.s.d.'s in distances, angles and torsion angles; correlations between e.s.d.'s in cell parameters are only used when they are defined by crystal symmetry. An approximate (isotropic) treatment of cell e.s.d.'s is used for estimating e.s.d.'s involving l.s. planes.
Refinement. Refinement of *F*^2^ against ALL reflections. The weighted *R*-factor *wR* and goodness of fit *S* are based on *F*^2^, conventional *R*-factors *R* are based on *F*, with *F* set to zero for negative *F*^2^. The threshold expression of *F*^2^ > σ(*F*^2^) is used only for calculating *R*-factors(gt) *etc*. and is not relevant to the choice of reflections for refinement. *R*-factors based on *F*^2^ are statistically about twice as large as those based on *F*, and *R*- factors based on ALL data will be even larger.

### Fractional atomic coordinates and isotropic or equivalent isotropic displacement parameters (Å^2^)

**Table:**

	*x*	*y*	*z*	*U*_iso_*/*U*_eq_	Occ. (<1)
O1A	0.3252 (8)	0.1907 (8)	0.2855 (6)	0.0247 (12)	0.539 (9)
O2A	0.7200 (11)	0.4559 (10)	0.3552 (8)	0.0263 (15)	0.539 (9)
O3A	0.0607 (10)	−0.0275 (11)	0.3127 (8)	0.0353 (18)	0.539 (9)
O4A	0.2823 (12)	−0.0290 (13)	0.1606 (10)	0.029 (2)	0.539 (9)
N1A	0.188 (3)	0.009 (3)	0.2706 (15)	0.025 (3)	0.539 (9)
C1A	0.1954 (16)	0.1367 (19)	0.3437 (13)	0.024 (2)	0.539 (9)
C2A	0.0942 (13)	0.2160 (16)	0.4548 (12)	0.024 (2)	0.539 (9)
H2AA	−0.0059	0.2062	0.5033	0.029*	0.539 (9)
C3A	0.1735 (15)	0.3187 (18)	0.4824 (14)	0.026 (3)	0.539 (9)
H3AA	0.1409	0.3841	0.5579	0.031*	0.539 (9)
C5A	0.3058 (10)	0.3035 (9)	0.3781 (8)	0.0256 (15)	0.539 (9)
C8A	0.7314 (16)	0.3690 (17)	0.2649 (12)	0.028 (2)	0.539 (9)
C9A	0.8760 (16)	0.322 (2)	0.1492 (12)	0.024 (3)	0.539 (9)
C10A	1.009 (2)	0.374 (4)	0.148 (2)	0.023 (3)	0.539 (9)
H10A	1.0027	0.4368	0.2193	0.028*	0.539 (9)
C11A	1.1495 (18)	0.334 (2)	0.0434 (16)	0.027 (2)	0.539 (9)
H11A	1.2350	0.3720	0.0449	0.032*	0.539 (9)
C12A	1.1681 (14)	0.2394 (19)	−0.0645 (13)	0.027 (2)	0.539 (9)
C13A	1.0316 (14)	0.1904 (19)	−0.0648 (12)	0.028 (3)	0.539 (9)
H13A	1.0379	0.1293	−0.1364	0.033*	0.539 (9)
C14A	0.8896 (14)	0.2312 (18)	0.0384 (11)	0.031 (3)	0.539 (9)
H14A	0.8015	0.1982	0.0348	0.037*	0.539 (9)
C15A	1.3292 (18)	0.177 (2)	−0.1713 (17)	0.049 (4)	0.539 (9)
H15A	1.4031	0.2314	−0.1629	0.073*	0.539 (9)
H15B	1.3048	0.1948	−0.2561	0.073*	0.539 (9)
H15C	1.3823	0.0660	−0.1629	0.073*	0.539 (9)
Br1A	0.6688 (5)	0.1302 (5)	0.4219 (5)	0.0395 (7)	0.539 (9)
Br2A	0.3626 (5)	0.5504 (4)	0.2175 (5)	0.0414 (6)	0.539 (9)
C6A	0.4234 (8)	0.3896 (8)	0.3487 (7)	0.0309 (16)	0.539 (9)
H6AA	0.4189	0.4424	0.4301	0.037*	0.539 (9)
C7A	0.5995 (9)	0.2863 (9)	0.2889 (7)	0.0312 (15)	0.539 (9)
H7AA	0.6066	0.2322	0.2074	0.037*	0.539 (9)
O1B	0.3468 (10)	0.1563 (9)	0.3082 (8)	0.024 (2)*	0.461 (9)
O2B	0.6979 (14)	0.4869 (11)	0.3410 (10)	0.022 (2)*	0.461 (9)
O3B	0.1011 (13)	−0.0594 (12)	0.2947 (11)	0.037 (3)*	0.461 (9)
O4B	0.3032 (19)	−0.0313 (19)	0.1354 (13)	0.037 (3)*	0.461 (9)
N1B	0.197 (4)	0.012 (4)	0.241 (2)	0.025 (3)	0.461 (9)
C1B	0.206 (2)	0.122 (2)	0.3321 (16)	0.025 (3)*	0.461 (9)
C2B	0.1002 (19)	0.194 (2)	0.4467 (16)	0.030 (3)*	0.461 (9)
H2BA	0.0011	0.1790	0.4910	0.036*	0.461 (9)
C3B	0.171 (2)	0.298 (2)	0.4858 (17)	0.031 (4)*	0.461 (9)
H3BA	0.1208	0.3734	0.5552	0.037*	0.461 (9)
C5B	0.3271 (12)	0.2658 (10)	0.4029 (9)	0.021 (2)*	0.461 (9)
C8B	0.713 (2)	0.393 (2)	0.2559 (14)	0.025 (3)*	0.461 (9)
C9B	0.860 (2)	0.340 (3)	0.1418 (16)	0.025 (4)*	0.461 (9)
C10B	1.003 (3)	0.374 (5)	0.145 (3)	0.030 (5)*	0.461 (9)
H10B	1.0002	0.4344	0.2156	0.036*	0.461 (9)
C11B	1.149 (2)	0.320 (3)	0.0433 (19)	0.029 (3)*	0.461 (9)
H11B	1.2444	0.3393	0.0480	0.035*	0.461 (9)
C12B	1.1500 (17)	0.235 (2)	−0.0665 (15)	0.026 (3)*	0.461 (9)
C13B	1.0072 (18)	0.203 (2)	−0.0705 (16)	0.029 (4)*	0.461 (9)
H13B	1.0071	0.1493	−0.1440	0.035*	0.461 (9)
C14B	0.8655 (18)	0.252 (2)	0.0331 (14)	0.028 (3)*	0.461 (9)
H14B	0.7728	0.2254	0.0308	0.033*	0.461 (9)
C15B	1.306 (2)	0.188 (2)	−0.1803 (17)	0.028 (3)*	0.461 (9)
H15D	1.4029	0.1346	−0.1477	0.043*	0.461 (9)
H15E	1.3172	0.2816	−0.2225	0.043*	0.461 (9)
H15F	1.2962	0.1196	−0.2428	0.043*	0.461 (9)
Br1B	0.6416 (5)	0.1406 (5)	0.4607 (4)	0.0297 (5)	0.461 (9)
Br2B	0.4087 (6)	0.5267 (6)	0.1895 (6)	0.0438 (8)	0.461 (9)
C6B	0.4744 (8)	0.3165 (8)	0.3932 (6)	0.0174 (16)*	0.461 (9)
H6BA	0.4387	0.4117	0.4505	0.021*	0.461 (9)
C7B	0.5614 (9)	0.3472 (9)	0.2554 (7)	0.0208 (17)*	0.461 (9)
H7BA	0.5963	0.2535	0.1965	0.025*	0.461 (9)

### Atomic displacement parameters (Å^2^)

**Table:**

	*U*^11^	*U*^22^	*U*^33^	*U*^12^	*U*^13^	*U*^23^
O1A	0.025 (3)	0.024 (3)	0.035 (3)	−0.015 (2)	−0.015 (2)	0.000 (2)
O2A	0.030 (4)	0.024 (3)	0.033 (3)	−0.015 (3)	−0.014 (3)	0.002 (3)
O3A	0.028 (4)	0.044 (4)	0.044 (4)	−0.029 (4)	0.000 (3)	−0.015 (3)
O4A	0.025 (4)	0.040 (4)	0.024 (5)	−0.018 (3)	0.000 (3)	−0.017 (3)
N1A	0.035 (3)	0.0307 (16)	0.021 (7)	−0.0187 (17)	−0.020 (4)	0.008 (5)
C1A	0.021 (3)	0.025 (4)	0.038 (4)	−0.015 (3)	−0.017 (2)	−0.001 (2)
C2A	0.021 (3)	0.025 (4)	0.038 (4)	−0.015 (3)	−0.017 (2)	−0.001 (2)
C3A	0.024 (4)	0.021 (5)	0.041 (4)	−0.016 (3)	−0.013 (3)	−0.004 (3)
C5A	0.030 (3)	0.022 (3)	0.033 (4)	−0.014 (3)	−0.014 (3)	0.003 (3)
C8A	0.024 (4)	0.028 (5)	0.043 (4)	−0.016 (4)	−0.016 (3)	0.000 (4)
C9A	0.025 (4)	0.025 (5)	0.029 (4)	−0.014 (4)	−0.014 (3)	0.001 (3)
C10A	0.030 (4)	0.027 (4)	0.026 (4)	−0.018 (3)	−0.018 (3)	0.004 (2)
C11A	0.025 (3)	0.026 (3)	0.037 (3)	−0.013 (2)	−0.015 (2)	0.005 (2)
C12A	0.025 (3)	0.026 (3)	0.037 (3)	−0.013 (2)	−0.015 (2)	0.005 (2)
C13A	0.027 (4)	0.030 (5)	0.030 (4)	−0.008 (4)	−0.016 (3)	−0.009 (3)
C14A	0.025 (4)	0.037 (6)	0.044 (4)	−0.017 (4)	−0.021 (3)	−0.005 (4)
C15A	0.033 (6)	0.059 (7)	0.044 (6)	−0.010 (5)	0.001 (5)	−0.020 (5)
Br1A	0.0302 (12)	0.0365 (8)	0.0632 (18)	−0.0172 (8)	−0.0264 (13)	0.0195 (12)
Br2A	0.0509 (16)	0.0327 (8)	0.0597 (17)	−0.0246 (10)	−0.0357 (13)	0.0189 (8)
C6A	0.033 (3)	0.031 (3)	0.037 (4)	−0.017 (3)	−0.017 (3)	0.002 (3)
C7A	0.031 (3)	0.030 (3)	0.039 (4)	−0.017 (3)	−0.012 (3)	0.001 (3)
N1B	0.035 (3)	0.0307 (16)	0.021 (7)	−0.0187 (17)	−0.020 (4)	0.008 (5)
Br1B	0.0228 (10)	0.0363 (6)	0.0371 (12)	−0.0145 (7)	−0.0151 (9)	0.0096 (9)
Br2B	0.0455 (16)	0.0513 (16)	0.0555 (18)	−0.0310 (13)	−0.0328 (14)	0.0264 (12)

### Geometric parameters (Å, °)

**Table:**

O1A—C5A	1.375 (7)	O1B—C1B	1.343 (10)
O1A—C1A	1.387 (8)	O1B—C5B	1.368 (9)
O2A—C8A	1.218 (8)	O2B—C8B	1.207 (11)
O3A—N1A	1.243 (10)	O3B—N1B	1.251 (12)
O4A—N1A	1.222 (9)	O4B—N1B	1.228 (11)
N1A—C1A	1.439 (9)	N1B—C1B	1.436 (11)
C1A—C2A	1.329 (9)	C1B—C2B	1.347 (10)
C2A—C3A	1.416 (9)	C2B—C3B	1.413 (11)
C2A—H2AA	0.9300	C2B—H2BA	0.9300
C3A—C5A	1.352 (9)	C3B—C5B	1.364 (11)
C3A—H3AA	0.9300	C3B—H3BA	0.9300
C5A—C6A	1.465 (9)	C5B—C6B	1.496 (11)
C8A—C9A	1.469 (9)	C8B—C9B	1.472 (10)
C8A—C7A	1.550 (13)	C8B—C7B	1.527 (18)
C9A—C10A	1.405 (9)	C9B—C10B	1.399 (11)
C9A—C14A	1.406 (8)	C9B—C14B	1.401 (11)
C10A—C11A	1.380 (9)	C10B—C11B	1.391 (11)
C10A—H10A	0.9300	C10B—H10B	0.9300
C11A—C12A	1.390 (9)	C11B—C12B	1.406 (10)
C11A—H11A	0.9300	C11B—H11B	0.9300
C12A—C13A	1.416 (10)	C12B—C13B	1.388 (11)
C12A—C15A	1.508 (9)	C12B—C15B	1.515 (10)
C13A—C14A	1.377 (9)	C13B—C14B	1.379 (11)
C13A—H13A	0.9300	C13B—H13B	0.9300
C14A—H14A	0.9300	C14B—H14B	0.9300
C15A—H15A	0.9600	C15B—H15D	0.9600
C15A—H15B	0.9600	C15B—H15E	0.9600
C15A—H15C	0.9600	C15B—H15F	0.9600
Br1A—C7A	1.992 (10)	Br1B—C6B	1.994 (8)
Br2A—C6A	1.987 (8)	Br2B—C7B	1.938 (10)
C6A—C7A	1.486 (10)	C6B—C7B	1.520 (10)
C6A—H6AA	0.9800	C6B—H6BA	0.9800
C7A—H7AA	0.9800	C7B—H7BA	0.9800
			
C5A—O1A—C1A	101.7 (6)	O3B—N1B—C1B	111.7 (12)
O4A—N1A—O3A	124.4 (10)	O1B—C1B—C2B	110.1 (8)
O4A—N1A—C1A	118.0 (9)	O1B—C1B—N1B	117.5 (10)
O3A—N1A—C1A	115.5 (10)	C2B—C1B—N1B	132.3 (10)
C2A—C1A—O1A	114.5 (7)	C1B—C2B—C3B	105.8 (9)
C2A—C1A—N1A	130.6 (8)	C1B—C2B—H2BA	127.1
O1A—C1A—N1A	114.8 (8)	C3B—C2B—H2BA	127.1
C1A—C2A—C3A	104.2 (7)	C5B—C3B—C2B	107.5 (10)
C1A—C2A—H2AA	127.9	C5B—C3B—H3BA	126.3
C3A—C2A—H2AA	127.9	C2B—C3B—H3BA	126.3
C5A—C3A—C2A	106.9 (7)	C3B—C5B—O1B	107.6 (8)
C5A—C3A—H3AA	126.5	C3B—C5B—C6B	136.7 (9)
C2A—C3A—H3AA	126.5	O1B—C5B—C6B	115.8 (7)
C3A—C5A—O1A	112.1 (6)	O2B—C8B—C9B	123.2 (13)
C3A—C5A—C6A	130.6 (7)	O2B—C8B—C7B	118.0 (12)
O1A—C5A—C6A	117.3 (6)	C9B—C8B—C7B	118.1 (10)
O2A—C8A—C9A	122.9 (9)	C10B—C9B—C14B	118.6 (10)
O2A—C8A—C7A	115.9 (9)	C10B—C9B—C8B	118.5 (11)
C9A—C8A—C7A	120.4 (8)	C14B—C9B—C8B	122.9 (11)
C10A—C9A—C14A	117.7 (8)	C11B—C10B—C9B	121.1 (12)
C10A—C9A—C8A	117.3 (8)	C11B—C10B—H10B	119.5
C14A—C9A—C8A	125.0 (9)	C9B—C10B—H10B	119.5
C11A—C10A—C9A	120.5 (9)	C10B—C11B—C12B	119.3 (12)
C11A—C10A—H10A	119.7	C10B—C11B—H11B	120.3
C9A—C10A—H10A	119.7	C12B—C11B—H11B	120.3
C10A—C11A—C12A	122.6 (9)	C13B—C12B—C11B	119.5 (10)
C10A—C11A—H11A	118.7	C13B—C12B—C15B	122.3 (11)
C12A—C11A—H11A	118.7	C11B—C12B—C15B	118.1 (10)
C11A—C12A—C13A	116.6 (8)	C14B—C13B—C12B	120.8 (12)
C11A—C12A—C15A	123.1 (10)	C14B—C13B—H13B	119.6
C13A—C12A—C15A	120.1 (10)	C12B—C13B—H13B	119.6
C14A—C13A—C12A	121.6 (8)	C13B—C14B—C9B	120.6 (12)
C14A—C13A—H13A	119.2	C13B—C14B—H14B	119.7
C12A—C13A—H13A	119.2	C9B—C14B—H14B	119.7
C13A—C14A—C9A	120.9 (8)	C12B—C15B—H15D	109.5
C13A—C14A—H14A	119.5	C12B—C15B—H15E	109.5
C9A—C14A—H14A	119.5	H15D—C15B—H15E	109.5
C5A—C6A—C7A	113.1 (6)	C12B—C15B—H15F	109.5
C5A—C6A—Br2A	110.2 (5)	H15D—C15B—H15F	109.5
C7A—C6A—Br2A	105.6 (6)	H15E—C15B—H15F	109.5
C5A—C6A—H6AA	109.3	C5B—C6B—C7B	115.9 (6)
C7A—C6A—H6AA	109.3	C5B—C6B—Br1B	106.1 (5)
Br2A—C6A—H6AA	109.3	C7B—C6B—Br1B	107.6 (5)
C6A—C7A—C8A	115.3 (7)	C5B—C6B—H6BA	109.0
C6A—C7A—Br1A	106.7 (5)	C7B—C6B—H6BA	109.0
C8A—C7A—Br1A	102.9 (7)	Br1B—C6B—H6BA	109.0
C6A—C7A—H7AA	110.5	C6B—C7B—C8B	111.7 (8)
C8A—C7A—H7AA	110.5	C6B—C7B—Br2B	109.3 (6)
Br1A—C7A—H7AA	110.5	C8B—C7B—Br2B	105.6 (8)
C1B—O1B—C5B	108.3 (8)	C6B—C7B—H7BA	110.0
O4B—N1B—O3B	124.1 (14)	C8B—C7B—H7BA	110.0
O4B—N1B—C1B	121.7 (12)	Br2B—C7B—H7BA	110.0
			
C5A—O1A—C1A—C2A	5.5 (14)	C5B—O1B—C1B—C2B	−5.7 (17)
C5A—O1A—C1A—N1A	−177.3 (19)	C5B—O1B—C1B—N1B	178 (2)
O4A—N1A—C1A—C2A	169 (2)	O4B—N1B—C1B—O1B	−10 (5)
O3A—N1A—C1A—C2A	5(4)	O3B—N1B—C1B—O1B	153 (2)
O4A—N1A—C1A—O1A	−7(4)	O4B—N1B—C1B—C2B	175 (2)
O3A—N1A—C1A—O1A	−172 (2)	O3B—N1B—C1B—C2B	−23 (5)
O1A—C1A—C2A—C3A	−7.5 (16)	O1B—C1B—C2B—C3B	8.7 (19)
N1A—C1A—C2A—C3A	176 (3)	N1B—C1B—C2B—C3B	−175 (3)
C1A—C2A—C3A—C5A	6.3 (17)	C1B—C2B—C3B—C5B	−8(2)
C2A—C3A—C5A—O1A	−3.3 (17)	C2B—C3B—C5B—O1B	5(2)
C2A—C3A—C5A—C6A	174.7 (11)	C2B—C3B—C5B—C6B	−173.2 (13)
C1A—O1A—C5A—C3A	−1.1 (14)	C1B—O1B—C5B—C3B	0.1 (17)
C1A—O1A—C5A—C6A	−179.3 (11)	C1B—O1B—C5B—C6B	178.9 (13)
O2A—C8A—C9A—C10A	3(3)	O2B—C8B—C9B—C10B	13 (4)
C7A—C8A—C9A—C10A	−166 (2)	C7B—C8B—C9B—C10B	−176 (3)
O2A—C8A—C9A—C14A	−175.3 (16)	O2B—C8B—C9B—C14B	−170 (2)
C7A—C8A—C9A—C14A	15 (3)	C7B—C8B—C9B—C14B	1(3)
C14A—C9A—C10A—C11A	−2(4)	C14B—C9B—C10B—C11B	−1(5)
C8A—C9A—C10A—C11A	180 (2)	C8B—C9B—C10B—C11B	176 (3)
C9A—C10A—C11A—C12A	−1(4)	C9B—C10B—C11B—C12B	3(5)
C10A—C11A—C12A—C13A	3(3)	C10B—C11B—C12B—C13B	−1(4)
C10A—C11A—C12A—C15A	−172 (3)	C10B—C11B—C12B—C15B	175 (3)
C11A—C12A—C13A—C14A	−2(2)	C11B—C12B—C13B—C14B	−1(3)
C15A—C12A—C13A—C14A	172.9 (17)	C15B—C12B—C13B—C14B	−177.8 (19)
C12A—C13A—C14A—C9A	−1(3)	C12B—C13B—C14B—C9B	3(3)
C10A—C9A—C14A—C13A	2(3)	C10B—C9B—C14B—C13B	−2(4)
C8A—C9A—C14A—C13A	−178.9 (17)	C8B—C9B—C14B—C13B	−179 (2)
C3A—C5A—C6A—C7A	140.5 (14)	C3B—C5B—C6B—C7B	−138.9 (18)
O1A—C5A—C6A—C7A	−41.6 (10)	O1B—C5B—C6B—C7B	42.8 (10)
C3A—C5A—C6A—Br2A	−101.6 (14)	C3B—C5B—C6B—Br1B	101.8 (18)
O1A—C5A—C6A—Br2A	76.3 (8)	O1B—C5B—C6B—Br1B	−76.5 (8)
C5A—C6A—C7A—C8A	−175.9 (8)	C5B—C6B—C7B—C8B	−178.2 (9)
Br2A—C6A—C7A—C8A	63.6 (8)	Br1B—C6B—C7B—C8B	−59.7 (9)
C5A—C6A—C7A—Br1A	−62.3 (6)	C5B—C6B—C7B—Br2B	65.3 (7)
Br2A—C6A—C7A—Br1A	177.1 (3)	Br1B—C6B—C7B—Br2B	−176.3 (4)
O2A—C8A—C7A—C6A	45.1 (16)	O2B—C8B—C7B—C6B	−43.9 (18)
C9A—C8A—C7A—C6A	−144.9 (13)	C9B—C8B—C7B—C6B	145.4 (16)
O2A—C8A—C7A—Br1A	−70.7 (13)	O2B—C8B—C7B—Br2B	74.8 (16)
C9A—C8A—C7A—Br1A	99.4 (14)	C9B—C8B—C7B—Br2B	−95.9 (17)

### Hydrogen-bond geometry (Å, °)

**Table:**

*D*—H···*A*	*D*—H	H···*A*	*D*···*A*	*D*—H···*A*
C2A—H2AA···O3A^i^	0.93	2.53	3.210 (15)	131
C3A—H3AA···O2A^ii^	0.93	2.51	3.216 (12)	133
C6A—H6AA···O2A^ii^	0.98	2.33	3.217 (10)	151
C13A—H13A···O3A^iii^	0.93	2.55	3.434 (13)	158

Symmetry codes: (i) −*x*, −*y*, −*z*+1; (ii) −*x*+1, −*y*+1, −*z*+1; (iii) −*x*+1, −*y*, −*z*.

## References

[bb1] Allen, F. H., Kennard, O., Watson, D. G., Brammer, L., Orpen, A. G. & Taylor, R. (1987). *J. Chem. Soc. Perkin Trans. 2*, pp. S1–19.

[bb2] Bernstein, J., Davis, R. E., Shimoni, L. & Chang, N.-L. (1995). *Angew. Chem. Int. Ed. Engl.* **34**, 1555–1573.

[bb3] Bruker (2009). *APEX2*, *SAINT* and *SADABS* Bruker AXS Inc., Madison, Wisconsin, USA.

[bb4] Cosier, J. & Glazer, A. M. (1986). *J. Appl. Cryst.* **19**, 105–107.

[bb5] Fun, H.-K., Shahani, T., Nithinchandra, & Kalluraya, B. (2010). *Acta Cryst.* E**66**, o2818–o2819. 10.1107/S1600536810040493 PMC3009087 21589008

[bb6] Hegde, J. C., Rai, G., Puranic, V. G. & Kalluraya, B. (2006). *Synth. Commun.* **36**, 1285–1290.

[bb7] Holla, B. S., Kalluraya, B. & Shridhar, K. R. (1986). *Curr. Sci.* **55**, 73–76.

[bb8] Holla, B. S., Kalluraya, B. & Shridhar, K. R. (1987). *Curr. Sci.* **56**, 236–238.

[bb9] Holla, B. S., Kalluraya, B. & Shridhar, K. R. (1992). *Rev. Roum. Chim.* **37**, 1159–1164.

[bb10] Rai, N. S., Kalluraya, B., Lingappa, B., Shenoy, S. & Puranic, V. G. (2008). *Eur. J. Med. Chem.* **43**, 1715–1720. 10.1016/j.ejmech.2007.08.002 17923171

[bb11] Sheldrick, G. M. (2008). *Acta Cryst.* A**64**, 112–122. 10.1107/S0108767307043930 18156677

[bb12] Spek, A. L. (2009). *Acta Cryst.* D**65**, 148–155. 10.1107/S090744490804362X PMC2631630 19171970

